# AI predicts a high risk in transplantation for myelofibrosis

**DOI:** 10.1182/blood.2025028816

**Published:** 2025-06-26

**Authors:** Simón Méndez-Ferrer

**Affiliations:** University of Seville, University of Cambridge and NHS Blood and Transplant

## Abstract

Allogeneic hematopoietic cell transplantation (allo-HCT) remains to be the only curative treatment for myelofibrosis (MF) but is associated with significant toxicity; it is therefore crucial to identify high-risk patients who might benefit from alternative therapies. In this issue of Blood, Hernández-Boluda et al^1^. develop an improved machine-learning-based model and web-based application to predict high risk of allo-HCT for the treatment of MF^1^.

MF is a reactive bone marrow (BM) scarring condition resulting from chronic inflammation in different hematological diseases, including myeloproliferative neoplasms (MPNs). MPN patients can present with MF at diagnosis (primary MF, pMF) or may acquire secondary MF (sMF) following disease evolution or transformation from polycythemia vera (PV) and, less frequently, essential thrombocythemia (ET)^2^. The different allele burden, loss of heterozygosis, acquisition of disease-modifying mutations and heterogenous interactions with specialized BM niches may all contribute to the disparate risk of sMF in different MPNs^2,3^.

While the management of MF in MPN has improved over the years, the main benefits of current treatments concern constitutional symptoms and overall quality of life, but do not significantly impact the allele burden or the risk to acquire secondary acute myeloid leukemia^2^. As a result, the only curative treatment for MF remains allo-HCT, which is only recommended in a minority of patients due to its toxicity^4^. Given the development of alternative treatments and numerous experimental agents currently being tested in clinical studies, it is imperative to improve allo-HCT outcome prediction, manage risk/benefit and better identify high-risk patients who might rather benefit from alternative therapies^4^.

Existing prognostic models for overall survival (OS) in MF after allo-HSCT have been key for the prognosis of non-relapse mortality (NRM) and clinical treatment decisions^4,5^. Currently, the identification of MF patients eligible for HCT relies on several criteria. One is high-molecular-risk profile, which can be scored in pMF using systems, such as the Mutation-Enhanced International Prognostic Score Systems MIPSS70^6^ (including mutations in ASXL1, EZH2, SRSF2, IDH1/ 2), which has been refined to MIPSS70+ and MIPSS70+ v.2.0, which includes the U2AF1Q157 variant as an additional high-molecular-risk mutation^7^. In sMF, the Myelofibrosis Secondary to PV and ET-Prognostic Model (MYSEC-PM) identifies 4 different risk categories^8^. Particularly, the Myelofibrosis Transplant Scoring System (MTSS) was developed to more precisely predict outcomes of HCT in pMF or sMF^5^. MTSS considers HCT-specific risk factors, including donor source and Karnofsky index or the Dynamic International Prognostic Scoring System (DIPSS/DIPSS+)^9^.

Recently, a panel of MF experts representing the European Society for Blood and Marrow Transplantation (EBMT) and the European Leukemia-Net (ELN) revised the previous recommendations for HCT in MF. These experts advised that higher-risk patients, determined by specific risk scores (DIPSS and MIPSS70/MIPSS70+), should undergo transplant assessment if they exhibit a low or moderate risk of NRM according to the MTSS tool. Additionally, intermediate-risk patients can also be considered for HCT if they have a low HCT-related risk^4^ (Table 1, left).

In this issue of Blood, Hernández-Boluda et al^1^. endeavored to better predict the risk of HCT in MF patients. To that aim, the authors analyzed data from 5,183 MF patients who underwent first allo-HCT between 2005 and 2020 at EBMT centers. The investigators built on the previous models and incorporated other variables in the prognostic model, such as patient co-morbidities and new therapeutic strategies.

The authors used machine learning approaches, including a Random Survival Forests (RSF), Oblique Random Survival Forests, XGBoost-based survival modeling (via the XGBSE library) and a deep learning method (DeepSurv). The cohort was divided into a training set (75%) and a test set (25%). The machine learning models were developed based on 10 key variables identified, comprising patient age, comorbidity index (HCT-CI), performance status, blood blasts, hemoglobin, leukocytes, platelets, donor type, conditioning intensity, and graft-versus-host disease prophylaxis (Table 1, right). The investigators found that RSF predicts OS and NRM similarly or better than the alternative techniques. While the same imputation methods were not consistently applied across all machine learning models (which might limit the analysis and the assumptions of the RSF), overall the RSF model appears to outcompete the other models identifying high-risk patients.

Compared with a four-level Cox regression-based score and other machine learning-based models, the RSF model provided better separation and identification of the high-risk group. While traditional performance metrics, such as the c-index, showed only modest differences overall, the performance of RSF was particularly enhanced compared to the one currently used in clinical practice based on the Center for International Blood and Marrow Transplant Research (CIBMTR) score^10^. The CIBMTR model does not account for haploidentical transplants or post-transplant cyclophosphamide use—both of which are increasingly common in HCT centers. The inclusion of these factors underscores the clinical relevance of the RSF model.

The main difference between the CIBMTR and RFS models is the relative risk assigned to patient populations. While the CIBMTR model classifies 40% of patients as low risk, 52% as intermediate risk, and only 8% as high risk, in the RSF model 25% of patients are classified as low risk, 50% as intermediate risk, and as many as 25% as high risk. Therefore, while the RSF does not separate intermediate risk groups over other approaches, it does identify a much larger proportion of high-risk patients, comprising ≈35% NRM rate and ≈40% overall mortality at 1 year. The authors observed that drop-off in survival outcomes for the highest-risk group is primarily driven by well-established prognostic factors, including older age, worse performance status, and hematologic markers of advanced disease. These variables are strongly linked to post-HCT mortality, leading to enhanced stratification at the higher end of the risk spectrum. While the RSF model does not aim to provide a mechanistic explanation of disease progression, it effectively captures the combined influence of these clinical factors on HCT outcomes.

Finally, a web application (https://gemfin.click/ebmt) emerging from the RSF model offers a practical tool to identify patients at high risk of poor outcome after allo-HCT, to better guide treatment decisions. The web-based calculator allows the identification of a subset of very high-risk patients with a predicted 1-year OS of <50%, prompting the consideration of other tailored therapeutic interventions.

## Figures and Tables

**Table 1:** Current recommendations for the indication of HCT for the treatment of MF patients and proposed variables to assess the risk. Left, Current recommendations for MF patient selection for allo-HCT according to a panel of MF experts representing the EBMT and ELN.^4^ Right, Key parameters proposed to stratify the risk of allo-HCT in MF^1^.

Current MF patient selection for allo-HCT^4^
pMF and intermediate-2/high-risk DIPSS or high-risk MIPSS70+ and low/intermediate MTSS
sMF and intermediate-2/high-risk sMF prognostic model score
pMF and intermediate-1 DIPSS or intermediate MIPSS70+ with low MTSS and a balance of patient preferences and treatment options
Patients >70 yr on an individual basis
**Parameters for consideration in proposed allo-HCT risk stratifier^1^**
Age
HCT-CI Score
Karnofsky Performance Status
Leukocytes (10^9/L)
Blood Blasts (%)
Hemoglobin (g/dL)
Platelets (10^9/L)
Donor Type
Type of conditioning
Type of GVHD prophylaxis
